# Sensory Organization Test Conditions Influence Postural Strategy Rather than Footwear or Workload

**DOI:** 10.3390/ijerph181910511

**Published:** 2021-10-07

**Authors:** Harish Chander, Sachini N. K. Kodithuwakku Arachchige, Alana J. Turner, Reuben F. Burch V, Jennifer C. Reneker, Adam C. Knight, Chip Wade, John C. Garner

**Affiliations:** 1 Neuromechanics Laboratory, Department of Kinesiology, Mississippi State University, Starkville, MS 39762, USA; snk128@msstate.edu (S.N.K.K.A.); ajt188@msstate.edu (A.J.T.); aknight@colled.msstate.edu (A.C.K.); 2 Human Factors & Athlete Engineering, Center for Advanced Vehicular Systems (CAVS), Mississippi State University, Starkville, MS 39759, USA; 3 Department of Industrial Systems and Engineering, Mississippi State University, Starkville, MS 39762, USA; burch@ise.msstate.edu; 4 Department of Population Health Science, John D. Bower School of Population Health, University of Mississippi Medical Center, Jackson, MS 39216, USA; jreneker@umc.edu; 5 Center for Diagnostics, Design, Device and Biomechanics, The University of Mississippi, Oxford, MS 38677, USA; cwade@olemiss.edu; 6 Department of Kinesiology and Health Promotion, Troy University, Troy, AL 36082, USA; jcgarner@troy.edu

**Keywords:** sensory organization test, postural strategy, occupational footwear, occupational workload

## Abstract

Background: Postural strategies such as ankle, hip, or combined ankle-hip strategies are used to maintain optimal postural stability, which can be influenced by the footwear type and physiological workload. Purpose: This paper reports previously unreported postural strategy scores during the six conditions of the sensory organization test (SOT). Methods: Fourteen healthy males (age: 23.6 ± 1.2 years; height: 181 ± 5.3 cm; mass: 89.2 ± 14.6 kg) were tested for postural strategy adopted during SOT in three types of occupational footwear (steel-toed work boot, tactical work boot, low-top work shoe) every 30 min during a 4-h simulated occupational workload. Postural strategy scores were analyzed using repeated measures analysis of variance at 0.05 alpha level. Results: Significant differences among postural strategy scores were only evident between SOT conditions, and but not between footwear type or the workload. Conclusions: Findings indicate that occupational footwear and occupational workload did not cause a significant change in reliance on postural strategies. The significant changes in postural strategy scores were due to the availability of accurate and/or conflicting sensory feedback during SOT conditions. In SOT conditions where all three types of sensory feedback was available, the ankle strategy was predominantly adopted, while more reliance on hip strategy occurred in conditions with absent or conflicting sensory feedback.

## 1. Introduction

In the event of an induced postural perturbation, the body’s postural control system selects and executes either one or a combination of postural strategies to provide counteracting measures to the postural perturbation, to regain postural stability and prevent falls. Postural strategies are usually classified as fixed support strategies which include ankle strategy, hip strategy, and a combination of ankle and hip strategy, and change in support strategies, which include stepping and grasping strategies [1,2,3]. While stepping and grasping strategies are usually resorted to only when the postural perturbation is large and causing the body’s center of mass (COM) to move outside of the base of support (BOS), ankle and hip strategies are more commonly used in static bilateral stance, with ankle strategy being selected with small perturbations and hip strategy being selected with larger perturbations [1,2]. Hence, the postural control system, through the selection and execution of appropriate postural strategies, helps in maintaining optimal postural stability and aid in preventing falls and fall-related injuries. Optimal postural stability is often seen as the task of maintaining upright balance during both static and dynamic activities, with minimal muscular and energy efficiency, without loss of balance, even in the presence of postural perturbations [1,2,3]. However, this optimal postural stability can vary among different populations, such as young, healthy, occupational, athletic, elderly, and clinical populations, further emphasizing the need for more research in this area.

The sensory organization test (SOT) on the Neurocom Equitest™ have been used extensively to measure and quantify postural stability [4,5,6,7], which uses the sway-referencing capabilities of the standing platform and visual surround to create six different sensory testing conditions (C1–C6) ranging from full availability of visual, vestibular, and somatosensory feedback, to absent and conflicting vision and somatosensory feedback [8]. Previous research has also demonstrated normative scores for SOT postural strategy scores (scores of 0–100; with scores towards 100 representing an ankle strategy, and scores away from 100 and towards 0, representing a hip strategy [8]), comparing the young, middle aged, and the elderly [5,6,7]. Significant differences were reported between young, middle, old, and elderly age groups, with postural strategy scores for the young being significantly representative of using ankle strategy, compared with other age groups [5,6]. Average postural strategy scores for young adults across the six SOT conditions from previous research demonstrate scores of C1: 96.7; C2: 96.3; C3: 97; C4: 92; C5: 86.4; C6: 86.4, respectively [6], with extremely similar scores for young healthy adults from another study [5]. However, these previous research studies have only compared SOT postural strategy scores among different age groups, while the impact of footwear and muscular fatigue due to physical workloads, and comparing postural strategy scores across SOT conditions, have not been conducted yet.

Several previous studies have reported on positive and negative influences of the type of footwear worn on postural stability, specifically among occupational footwear that is designed primarily for safety purposes rather than for optimal postural control [9,10,11,12,13]. Based on previous research, for optimal postural stability maintenance, occupational footwear, in addition to meeting safety standards, should be lighter in mass, have an elevated boot shaft, have thin and firm midsole, have a low heel height, and low heel-to-toe drop [14]. More specifically, the current research team have completed several studies and analyzed the impact of three types of occupational footwear (steel-toed work boots—ST; tactical work boots—TB; low-top slip-resistant shoes—LT) on various aspects of postural stability such as postural sway, postural response times, and muscle activity from lower extremity muscles, in non-workload conditions, as well as when exposed to both acute high-intensity and chronic low-intensity physiological workloads [15,16,17,18,19,20]. When significant differences were found between the footwear, the ST and TB were reported to have better postural stability compared with LT, owing to their design characteristics [15,17], and in other studies, no significant differences existed between the footwear [16,20]. Additionally, physical exertion due to the physiological workloads was more attributable to the demonstrated significantly decreased postural stability [15,17,18,19].

In one of the above-mentioned studies, the three types of occupational footwear (ST, TB, and LT) were assessed for postural stability when exposed to a low-intensity simulated occupational workload. The occupational workload consisted of 4 h of continuous walking and standing at a self-selected pace and self-selected path on a hard, firm surface. Participants were allowed to stop walking and spend a few minutes standing, but were never allowed to sit down and rest. This standing/walking for prolonged duration and testing in every 30-min intervals was adopted from previous studies attempting to simulate occupational workloads [15,21]. Postural stability was tested using the SOT, before the start of the workload and then every 30 min during the 4 h of workload. Findings from the study on the impact of these types of occupational footwear and occupational workload on postural stability were reported as center of pressure (COP)-derived postural sway variables [15], SOT equilibrium (EQ) scores [17], and as lower extremity muscle activity during SOT [20] (Chander et al., 2021a). Postural stability quantified by COP postural sway variables and SOT EQ scores indicated that ST and LT demonstrated significantly better postural stability compared with LT [15,17], and that lower extremity muscle activity was not impacted by these types of occupational footwear [20]. However, significant differences due to the workload over the 4 h duration were only detected by COP postural sway variables [15], but not by SOT EQ scores and lower extremity muscle activity during SOT [17,20].

Subsequently, the postural strategy used during maintenance of postural stability during the six different test conditions of the SOT which uses conflicting visual and somatosensory feedback has not been analyzed yet. The design features on the footwear, such as boot shaft height, can influence the postural strategy selected and used, as elevated boot shafts have been shown to restrict movement around the ankle joint [22,23]. Subsequently, fatigue caused by a physical workload has been shown to be detrimental in maintaining an upright stance, and the use of hip strategy is often required over ankle strategy to maintain optimal postural stability [24,25]. Therefore, the purpose of the study was to assess the impact of occupational footwear and occupational workload on postural strategy during the six conditions of the SOT. The specific aims of the study included: (i) to assess whether occupational footwear type and workload influence postural strategy during each of the six conditions of the SOT; (ii) to assess whether occupational footwear and SOT conditions influence postural strategy; and (iii) to assess whether occupational workload and SOT conditions influence postural strategy.

## 2. Materials and Methods

The current paper reports previously unreported postural strategy analysis from the occupational footwear and occupational workload study. Postural stability outcomes due to these occupational footwear and workload have been previously reported through COP-derived postural sway data [15], through SOT equilibrium scores [17], and through muscle activity during SOT [20].

### 2.1. Participants

Fourteen healthy adult males (age: 23.6 ± 1.2 years; height: 181 ± 5.3 cm; mass: 89.2 ± 14.6 kg) with no history of any orthopedic or neurological disorders completed the study. A total of 16 participants were recruited; one failed to complete the study, and one was considered an outlier who did not adhere to the testing requirements. Informed consent to participate in the study based on the University of Mississippi’s institutional review board (IRB)-approved protocol (IRB Protocol# 11-150—Approval Date: 02/17/2011) was obtained from participants.

### 2.2. Experimental Procedures

**Figure 1:**
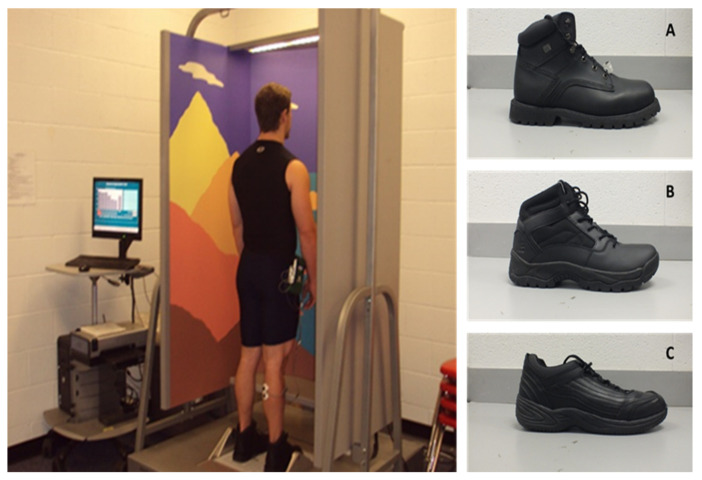
Sensory organization test (SOT) performed on the Neurocom Equitest™ with one of the types of occupational footwear tested (steel-toed work boot). (**A**) steel-toed work boot (ST); (**B**) tactical work boot (TB); (**C**) low-top slip-resistant shoe (LT).

After an initial familiarization day which also included completing anthropometric assessments, all participants were tested for postural stability using the SOT in three separate testing days (separated by 72 h to avoid undue fatigue), on all three types of occupational footwear (ST, TB, LT) (Figure 1) when exposed to a low-intensity simulated occupational workload. On each testing day, the participants wore one of the types of occupational footwear, which was randomly assigned, and the allocation footwear was counterbalanced between the participants. Participants began each testing day with a brief dynamic warm-up wearing the occupational footwear assigned for the day. Participants then completed the first pre-test of SOT on the Neurocom Equitest™, which uses its sway-referencing capabilities of the visual surround and force platforms to create six individual testing conditions with 3 trials of 20 s each (eyes open (EO), eyes closed (EC), eyes open sway referenced vision (EOSRV), eyes open sway referenced platform (EOSRP), eyes closed sway referenced platform (ECSRP) and eyes open sway referenced vision and platform (EOSRVP)). On completion, participants started the low-intensity workload which consisted of 4 h (240 min) of continuous walking at a self-selected pace and on a self-selected path on an even flat vinyl flooring, with postural stability measured every 30 min. Thus, a total of 9 testing measures was performed (0 min, 30 min, 60 min, 90 min, 120 min, 150 min, 180 min, 210 min, 240 min). The exact same procedures were repeated for the other two types of footwear on two separate days of testing, which marked the completion of the study.

### 2.3. Data and Statistical Analysis

Postural strategy scores were derived from the Neurocom Equitest™ (Neurocom International Inc. Clackamas, Oregon, USA) for each condition of the SOT. Scores ranged from 0 to 100, with scores closer to 100 indicating the use of ankle strategy, while scores away from 100 indicating the use of hip strategy in maintaining postural stability [8,26]. Postural strategy scores from three trials of each condition of the SOT were averaged and were used for statistical analyses. Within-subjects repeated measures analysis of variance (RM ANOVA) statistical analyses were used, based on the three specific aims of this study. For specific aim (i), a 3 (footwear) × 9 (time points of testing) within-subjects RM ANOVA was run for each SOT condition. If significant footwear × time interaction was found, a post hoc simple effects comparison with a Bonferroni correction was made, and if significant footwear existed, they were followed up with post hoc pairwise comparisons with a Bonferroni correction. If there were no significant footwear × time interaction or main effect significances were detected, two other RM ANOVAs were run. For specific aim (ii), a 3 (footwear) × 6 (SOT conditions) within-subjects RM ANOVA with all nine points of testing (0 min to 240 min) averaged was run, and for specific aim (iii), a 9 (time points of testing) × 6 (SOT conditions) within-subjects RM ANOVA with all footwear averaged was run. Similar post hoc comparisons were followed up for significant interaction or main effect significances. All statistical analyses were run using SPSS v27 at an alpha level of 0.05.

## 3. Results

### 3.1. Specific Aim (i)

The 3 (footwear) × 9 (time points of testing) RM ANOVA did not reveal any significant differences in the main effect for footwear or time points of testing when analyzed for each SOT condition, except for a single main effect significance for time points of testing during the ECSRP SOT condition [F (8, 104) = 3.204, *p* = 0.003, ƞ_p_^2^ = 0.198]. Post hoc pairwise comparison revealed that postural strategy score during the pre-test (0 min) was significantly lower compared with the 240th minute. With no other significant differences across any footwear or time point, for any other SOT condition individually, two further RM ANOVAs were performed with postural strategy scores for all time points of testing averaged and with postural strategy scores for all footwear averaged and analyzed against all six SOT conditions.

### 3.2. Specific Aim (ii)

**Figure 2:**
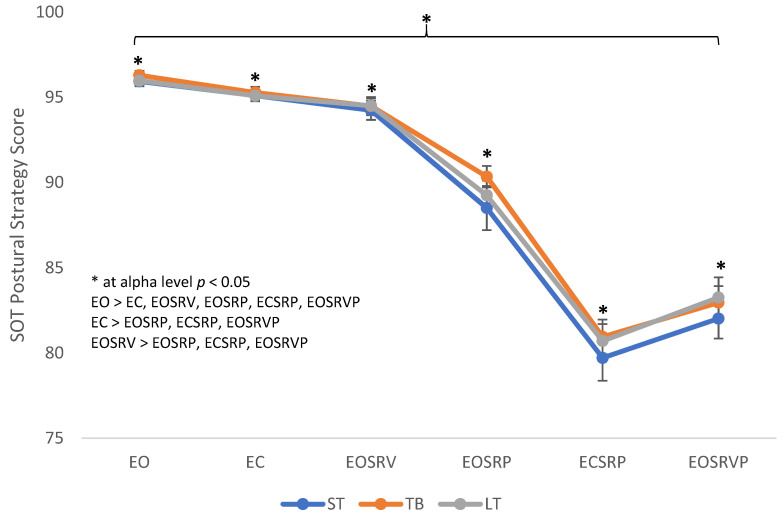
Sensory organization test (SOT) postural strategy scores for three types of footwear: steel-toed work boot (ST), tactical work boot (TB), and low top shoe (LT), during the six SOT conditions: eyes open (EO), eyes closed (EC), eyes open sway referenced vision (EOSRV), eyes open sway referenced platform (EOSRP), eyes closed sway referenced platform (ECSRP) and eyes open sway referenced vision and platform (EOSRVP). * indicates significant difference for SOT conditions. List within the graph explains the significant pairwise comparisons. Significant differences at *p* < 0.05 level; bars represent standard errors.

The 3 (footwear) × 6 (SOT conditions) RM ANOVA revealed significant main effect differences for SOT conditions [F (5, 65) = 217.206, *p* < 0.001, ƞ_p_^2^ = 0.944]. Post hoc pairwise comparisons revealed significant differences in postural strategy scores between SOT conditions [EO > EC (*p* < 0.001), EOSRV (*p* = 0.004), EOSRP (*p* < 0.001), ECSRP (*p* < 0.001), EOSRVP (*p* < 0.001); EC > EOSRP (*p* < 0.001), ECSRP (*p* < 0.001), EOSRVP (*p* < 0.001); EOSRV > EOSRP (*p* < 0.001), ECSRP (*p* < 0.001), EOSRVP (*p* < 0.001); EOSRP > ECSRP (*p* < 0.001); EOSRVP (*p* < 0.001)] (Figure 2). No other significant footwear main effect or footwear × SOT condition interaction was found.

### 3.3. Specific Aim (iii)

**Figure 3:**
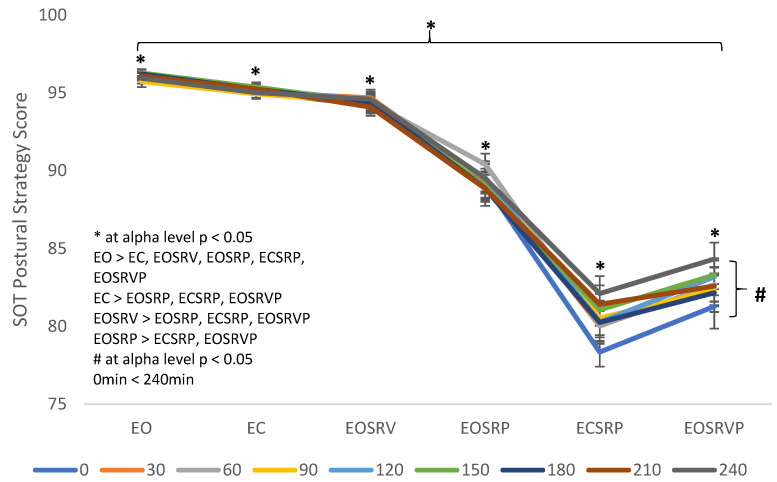
Sensory organization test (SOT) postural strategy scores for nine time points of testing: 0 min, 30 min, 60 min, 90 min, 120 min, 150 min, 180 min, 210 min, and 240 min, during the six SOT conditions: eyes open (EO), eyes closed (EC), eyes open sway referenced vision (EOSRV), eyes open sway referenced platform (EOSRP), eyes closed sway referenced platform (ECSRP) and eyes open sway referenced vision and platform (EOSRVP). * indicates significant difference for SOT conditions. # indicates significant difference between time points of testing. List within the graph explains the significant pairwise comparisons. Significant differences at *p* < 0.05 level; bars represent standard errors.

The 9 (time points of testing) × 6 (SOT conditions) RM ANOVA revealed a significant main effect for time points tested [F (8, 104) = 2.635, *p* = 0.011, ƞ_p_^2^ = 0.169] and significant main effect differences for SOT conditions [F (5, 65) = 215.795, *p* < 0.001, ƞ_p_^2^ = 0.943]. Post hoc pairwise comparisons revealed significant differences in postural strategy scores between time point of testing [0 min < 240 min (*p* = 0.036)] and between SOT conditions [EO > EC (*p* < 0.001), EOSRV (*p* = 0.004), EOSRP (*p* < 0.001), ECSRP (*p* < 0.001), EOSRVP (*p* < 0.001); EC > EOSRP (*p* < 0.001), ECSRP (*p* < 0.001), EOSRVP (*p* < 0.001); EOSRV > EOSRP (*p* < 0.001), ECSRP (*p* < 0.001), EOSRVP (*p* < 0.001); EOSRP > ECSRP (*p* < 0.001); EOSRVP (*p* < 0.001)] (Figure 3). No other significant footwear main effect or footwear × SOT condition interaction was found.

## 4. Discussion

The purpose of the study was to assess the impact of occupational footwear and occupational workload on postural strategy during the six conditions of the SOT. The specific aims of the study included (i) to assess whether occupational footwear type and workload influence postural strategy during each of the six conditions of the SOT, (ii) to assess whether occupational footwear and SOT conditions influence postural strategy, and (iii) to assess whether occupational workload and SOT conditions influence postural strategy. Overall, the occupational footwear or the occupational workload did not significantly influence postural strategy adoption, except for one significant time point of testing for only one SOT condition (ECSRP), which can be largely interpreted as a learning effect that accompanies with repeated testing on the SOT [27]. The type of postural strategy adopted or the reliance on one postural strategy more than the other (more ankle or more hip strategy) was influenced by the type of SOT condition tested, as each SOT condition forces to use different sensory systems to maintain postural stability due to the conflicting sensory information presented during the SOT. In SOT conditions which were less challenging, where all three visual, somatosensory, and vestibular sensory systems were available without conflicts, a significantly greater reliance on ankle strategy was observed, while significantly greater use of hip strategy was observed during SOT conditions where there was sensory conflict. However, even with prior findings from the current study demonstrating that occupational footwear and/or occupational workload significantly influenced postural stability based on COP postural sway variables [15], SOT EQ scores [17], and lower extremity EMG muscle activity [20], the current findings indicate that occupational footwear and occupational workload did not cause a significant change in reliance of ankle or hip strategy. Significant changes were only observed due to the type of SOT condition, attributing the shift from a predominant ankle strategy to relying less on the ankle strategy, and comparatively more reliance on hip strategy to maintain postural stability occurred rather than a complete shift to hip strategy, to maintain postural stability, due to the sensory feedback and sensory conflict inherent of the SOT conditions, rather than due to the type of occupational footwear or the simulated occupational workload.

While physical workload and muscular fatigue, especially of the lower extremity, have shown a greater reliance on hip strategy using stronger proximal muscles to minimize external perturbations and thereby to maintain balance [24,25,28], the current simulated occupational workload of 4 h of continuous walking at a self-selected pace and self-selected path did not contribute to a significant deviation from ankle strategy with greater reliance on hip strategy to maintain postural stability. Justification could be that unlike the previous studies [24,25,28], the physical workload was not a high-intensity fatiguing workload, but a simulated occupational low-intensity prolonged duration workload which, in the current study, did not influence the postural strategy adopted. Extensive literature compiled as systematic reviews on occupational footwear indicate their impact on postural stability and locomotion [12,18]. As such, the design features on these types of footwear, such as an elevated boot shaft, mass, heel height, heel-to-toe drop, mid-sole and insole hardness, etc., have been attributed to the impact on postural stability. For example, a design feature relevant to the current study is the boot shaft height, where footwear with above-ankle elevated boot shafts have been reported to provide proprioceptive feedback to enhance postural stability [18] and have also been reported to minimize ankle joint range of motion [23]. However, in the current study, even with differences in boot shaft height (above ankle: ST and TB; below ankle: LT), there were no significant differences in postural strategy, where one design feature could have compensated for another. Additionally, these same types of footwear have demonstrated significant differences in postural stability when quantified by COP postural sway variables [10]. However, postural strategy scores were not significantly influenced by occupational footwear type. This could be due to a couple of reasons, where the design features of the currently tested occupational footwear may not have been more pronounced to cause significant changes in postural strategy, and/or the low-intensity workload did not have a significant interaction with this occupational footwear to cause changes in the specific footwear at specific points of testing during the 4 h.

The SOT conditions significantly influenced the postural strategy adopted, where the availability and unavailability of accurate and/or conflicting sensory feedback, based on the type of SOT condition, can be attributed to the observed findings. In a normal upright bilateral stance, individuals predominantly use ankle strategy to maintain postural stability, while hip strategy is relied on during large postural perturbations [1,29]. The SOT conditions of EO (all three sensory feedback available) and EC (visual feedback absent) demonstrated a greater reliance on ankle strategy, including during EOSRV, even with conflicting visual feedback. However, with EOSRP (conflicting somatosensory feedback), ECSRP (conflicting somatosensory feedback with absent visual feedback), and EOSRVP (conflicting visual and somatosensory feedback), there was a significantly greater reliance on hip strategy. Thus, when maintaining postural stability was tasked under relatively easier environments (sensory systems predominantly available), there was a significantly greater reliance on ankle strategy, and when maintaining postural stability was tasked under relatively difficult environments (absent or predominantly conflicting sensory systems available), there was a shift towards utilizing more hip strategy.

More recently, the current research team also analyzed the impact of three different types of alternative recreational footwear (Crocs, flip-flops, Vibram five-toed shoes) and two different types of military footwear (standard tactical and minimalist tactical) on postural strategy during SOT, when exposed to low-intensity (self-selected pace, 1 mile walk) and high-intensity (a load-carriage treadmill run until volitional exhaustion) physical workloads, respectively [30]. The findings from the current study support this, as low-intensity workloads did not cause significantly greater reliance on hip strategy, whereas a high-intensity workload did [30]. In that same study, a significant footwear effect was evident where the standard tactical military boot adopted a great use of hip strategy compared with the minimalist military boot, suggesting that footwear design features can potentially impact the postural strategy adopted and that the minimalist military boot was better designed for optimal postural stability with a significantly lesser reliance on hip strategy [30]. However, in the current study, the occupational footwear did not influence postural strategy. Postural stability of these ST and TB types of occupational footwear under acute high-intensity workloads have also been previously studied, with the ST demonstrating greater postural stability and the high-intensity workload being detrimental to postural stability [18]. However, postural strategy selection and adoption donning this occupational footwear when exposed to high-intensity workload have not been conducted yet, and may be considered for future research. Based on previous studies assessing occupational footwear and occupational workloads [13,15] and based on results from this current analysis, using SOT postural strategy scores in isolation to assess postural stability is not recommended, and a combination of SOT equilibrium scores, SOT postural strategy scores, as well as COP-derived postural sway variables are recommended.

Limitations of the study include the testing of healthy young population with a healthy postural control system. However, this was done to test the impact of the footwear and the workload, free from any physiological or pathological impacts on the postural control system. Therefore, more research on elderly and clinical populations are much warranted. Additionally, the Neurocom Equitest™ does not have any clear cut-off thresholds for differentiating an exclusive ankle from an exclusive hip strategy, as the interpretation of the postural strategy scores is based on scores from 0 to 100, where more postural strategy scores closer to 100 indicate a predominant use of ankle strategy, and scores closer to 0 or further away from 100, indicate hip strategy. Hence, while the postural strategy scores from the Neurocom Equitest™ provide a basic understanding of the postural control system’s reliance on a particular strategy, it may not be a true representation of the strategy analyzed, selected, and adopted. Future studies also focus on sensory ratio analysis which is one of the functions of the Neurocom Equitest™, as they can provide further understanding of the sensory system selection and adoption in maintaining postural stability. However, these scores still provide crucial information on postural strategy selection and adoption, especially in environments where sensory feedback is limited or with sensory conflicts. These findings offer an understanding of the behavior of occupational footwear when exposed to simulated low-intensity occupational workload while maintaining optimal postural stability. The significant differences between different SOT conditions further offer an understanding of the postural strategies adopted when sensory feedback is readily available, absent, and/or conflicted. These findings can aid postural control training among healthy, occupational, athletic, geriatric, and clinical populations.

## 5. Conclusions

Based on the results from this study, and comparing postural stability findings from the same study from previously published literature [15,17], it is evident that while postural stability quantified by COP-derived postural sway variables and SOT EQ scores were influenced by the type of occupational footwear and workload, individuals did not have to significantly change postural strategy from a predominant ankle strategy to relying more on hip strategy to maintain postural stability. The significant changes in postural strategy scores were due to the availability and unavailability of accurate and/or conflicting sensory feedback, based on the type of SOT condition. SOT conditions where all three, visual, vestibular, and somatosensory feedback, were readily available, an ankle strategy was predominantly adopted, while less reliance on the ankle strategy with comparatively more reliance on hip strategy to maintain postural stability occurred in conditions with absent or conflicting sensory feedback. Hence, the SOT conditions rather than occupational footwear and workload influence the postural strategy adopted to maintain optimal postural stability.
